# Multi-arm U-Net with dense input and skip connectivity for T2 lesion segmentation in clinical trials of multiple sclerosis

**DOI:** 10.1038/s41598-023-31207-5

**Published:** 2023-03-13

**Authors:** Anitha Priya Krishnan, Zhuang Song, David Clayton, Xiaoming Jia, Alex de Crespigny, Richard A. D. Carano

**Affiliations:** 1 Data Analytics and Imaging, Pharma Personalized Healthcare, Genentech Inc., 600 E Grand Ave., South San Francisco, CA 94080 USA; 2 Clinical Imaging Group, gRED, Genentech Inc., South San Francisco, CA USA; 3 Translational Medicine OMNI – Biomarker Development, Genentech Inc., South San Francisco, CA USA

**Keywords:** Multiple sclerosis, Demyelinating diseases, Multiple sclerosis

## Abstract

T2 lesion quantification plays a crucial role in monitoring disease progression and evaluating treatment response in multiple sclerosis (MS). We developed a 3D, multi-arm U-Net for T2 lesion segmentation, which was trained on a large, multicenter clinical trial dataset of relapsing MS. We investigated its generalization to other relapsing and primary progressive MS clinical trial datasets, and to an external dataset from the MICCAI 2016 MS lesion segmentation challenge. Additionally, we assessed the model’s ability to reproduce the separation of T2 lesion volumes between treatment and control arms; and the association of baseline T2 lesion volumes with clinical disability scores compared with manual lesion annotations. The trained model achieved a mean dice coefficient of ≥ 0.66 and a lesion detection sensitivity of ≥ 0.72 across the internal test datasets. On the external test dataset, the model achieved a mean dice coefficient of 0.62, which is comparable to 0.59 from the best model in the challenge, and a lesion detection sensitivity of 0.68. Lesion detection performance was reduced for smaller lesions (≤ 30 μL, 3–10 voxels). The model successfully maintained the separation of the longitudinal changes in T2 lesion volumes between the treatment and control arms. Such tools could facilitate semi-automated MS lesion quantification; and reduce rater burden in clinical trials.

## Introduction

Multiple sclerosis (MS) is a chronic autoimmune disease accompanied by demyelination and atrophy of the brain and spinal cord. Quantification of lesions and atrophy assessments from magnetic resonance imaging (MRI) are used to evaluate disease activity and monitor disease progression in MS. T2 lesions are typically larger and occur more widely than T1 lesions, but are relatively nonspecific for MS. T2 lesion volume and the number of new and/or enlarging T2 lesions have been used as secondary endpoints in clinical trials of various disease-modifying therapies for MS. Currently, such assessments are performed manually by radiologists and have high inter-rater variability^1^.

Recently, multiple deep-learning models have been developed for segmenting T2 lesions, either alone^2–4^ or in combination with T1 lesions^5^ or other brain structures^6,7^, using small-sized (training set *n* ≤ 50 patients) to reasonably sized (training set *n* ≤ 500) datasets curated either at single^2,3,5^ or multiple sites^4,6,7^. With the various model architectures and different datasets used for training and evaluation, the main challenges lie in assessing the relative performance of the models and in ascertaining their generalization to unseen data that may have similar and/or shifts in their distributions. The diverse MRI acquisition protocols, scanner types, and the differences in lesion annotation styles used as ground truths (GTs) for training these models are the most common sources of variability. To address these challenges, two datasets have been made publicly available to the MS community: ISBI 2015, which includes longitudinal datasets from 19 MS patients with 4–5 timepoints each and GT delineations provided by two expert readers^8^; and MICCAI 2016, which includes MRI datasets from 53 patients acquired from four scanners and GT annotations provided by seven experts^9^.

Convolutional neural networks performed the best for MS T2 lesion segmentation in challenges with both the 2D and 3D U-Nets^10^, achieving top scores. The best performing model on the ISBI 2015 dataset was a 2D U-Net operating on a stack of neighboring slices (2.5D) from any orthogonal orientation^2^; a 3D U-Net trained on large input patches^5^ was also among the best performing models. For better extraction of lesion-related features from multimodal MRI, Aslani et al. developed a multibranch version of a 2D U-Net operating on 2.5D inputs from three orthogonal slices, with separate extraction of lesion features from the fluid-attenuated inversion recovery (FLAIR), T1-weighted (T1w), and T2-weighted (T2w) sequences^3^. However, the resulting model didn’t perform as well as the 2D U-Net^2^, probably due to overfitting of a larger network when trained on a small dataset. Rather than developing models on the small training set of these challenges, researchers have used larger internal datasets for model training and tested their generalization to the ISBI 2015 and/or MICCAI 2016 datasets^6,7^. In particular, McKinley et al.^7^ showed that the generalization to external datasets was improved by jointly segmenting T2 lesions with other brain structures.

Besides training on MRI datasets from clinical practice, T2 lesion segmentation models have also been developed using MRI datasets and annotations from clinical trials in MS using ‘leave-center-out’ approaches^11^. Although clinical trials result in large-scale MRI datasets and GT lesion annotations, they are acquired across multiple sites and scanners using homogenized MRI acquisition protocols and do not contain the greater protocol variability seen in real-world datasets (RWD). To the best of our knowledge, the generalization of models trained on clinical trial datasets to those in clinical practice has not been studied. In addition, most prior work has focused on T2 lesion segmentation and detection quality cross-sectionally. Brugnara et al. alluded to the longitudinal dynamics captured by the model in representative cases^5^. Studies looking into the longitudinal trends at the cohort level in clinical trials are currently lacking. On a related note, the T2 lesion secondary imaging endpoints in the OPERA trials were on the count of new and enlarging T2 lesions and the segmentation of these lesions is an active area of research, which is beyond the scope of the current work.

The objectives of this study were to develop a multi-arm, 3D U-Net for T2 lesion segmentation using MRI datasets and GT annotations from a clinical trial of relapsing MS (RMS) and to evaluate its generalization both to other trials of RMS and progressive MS with the same or similar acquisition protocols, and to high-quality MRI datasets from clinical practice. We hypothesized that the multi-arm, 3D U-Net architecture would enable better extraction of 3D features from multimodal MRI data with parallel flow of information across various depths/resolutions of the network; further, the large-scale training set will enable better generalization to datasets from other trials and from clinical practice with comparable image quality. We benchmarked the performance of our model on the ISBI 2015 dataset. In addition, we investigated the model’s ability to reproduce longitudinal T2 volume changes in the treatment and control arms at the cohort level, and the association of baseline T2 lesion volumes with outcomes identified from manual reads.

## Materials and methods

### MRI datasets and manual GT annotations

#### MS clinical trials

The proposed T2 lesion segmentation model was developed retrospectively using MRI datasets and GT lesion annotations from the OPERA I trial (NCT01247324, *n* = 821) investigating ocrelizumab in patients with RMS. The model was evaluated on data from the OPERA II (NCT01412333, *n* = 835, RMS) and ORATORIO (NCT01194570, *n* = 732, primary progressive MS [PPMS]) trials of ocrelizumab and the OLYMPUS (NCT00087529, *n* = 439, PPMS) trial of rituximab as the internal test sets. Details on patient selection, trial designs, MRI acquisition, clinical assessments, and outcomes were provided in the original reports^12–14^ of the respective trials. For each trial, research was performed in accordance with all relevant guidelines and regulations. Study protocols were approved by the relevant institutional review boards/ethics committees and all data were de-identified by each participating institution. All patients provided written informed consent and each trial was conducted in accordance with the International Conference on Harmonization guidelines for Good Clinical Practice and the Declaration of Helsinki. As the T2 lesion segmentation model used a multimodal input of T2w, T2w FLAIR, and T1w pre-contrast (T1p) images, the visits that contained these sequences and the corresponding GT annotations were included in this study.

Standard conventional brain MRI data, including 3D T1p (spoiled gradient echo; repetition time [TR]/echo time [TE]/flip angle: 28–30 ms/5–11 ms/27–30 degrees), 2D T2w (fast spin echo; TR/TE: 4–6.2 s/74–91 ms; echo train length [ETL]: 7–11), and T2w FLAIR (TR/TE: 9–10 s/66–100 ms; ETL: 8–12; inversion time: 2.2–2.5 s) were acquired at baseline; Weeks 24, 48, and 96 for the OPERA trials; and Weeks 24, 48, and 120 for the ORATORIO trial. The same MRI sequences were acquired at baseline and Weeks 48, 96, and 122 for the OLYMPUS trial, with a slightly modified acquisition protocol. All sequences were acquired axially with a voxel resolution of ~ 1 × 1 × 3 mm^3^. Lesion segmentations for all four studies were performed by the same central reading facility, providing a consistent set of GT annotations. It should be noted that the reading facility used for the original results of OLYMPUS was not the same as the facility used in this study, and therefore one has to be cautious when comparing lesion measurements in this report with the original. GT lesion annotations were obtained using a semi-automated approach, where the initial automatic segmentation was performed using a Bayesian classifier^15^, which was then verified/corrected by readers (single reads randomly assigned to 27 readers). Preprocessing included bias field correction^16^, rigid registration of baseline images to a Montreal Neurological Institute template, rigid registration of follow-up MRI to baseline^17^, and skull stripping^18^.

### ISBI 2015 dataset

The challenge dataset included proton density-weighted (PDw), T1p, T2w, and T2w FLAIR sequences acquired at a single site on a 3 T MRI scanner and GT reads provided by two experienced readers^8^. The training set consisted of longitudinal MRI datasets from five patients with 4–5 timepoints each (*n* = 19) and the test set included datasets of 14 patients with 4–6 timepoints each (*n* = 61). The in-plane resolution was ~ 0.8 mm for the T1p, T2w, and FLAIR sequences, with slice thicknesses of 1.17 mm for T1w and 2.2 mm for T2w and FLAIR MRI. The images were preprocessed and resampled to an isotropic, 1 mm resolution.

### MICCAI 2016 dataset

This dataset consisted of PDw, T1p, T1w post-contrast, T2w, and T2w FLAIR MRI of 53 patients with MS acquired on four different scanners using a harmonized imaging protocol^9^. GT T2 lesion annotations were provided by seven expert readers and the consensus mask was obtained using the logarithmic opinion pool based simultaneous truth and performance level estimation^19^. 3D FLAIR and T1w MRI were acquired with 0.43–1 mm in-plane resolution and a slice thickness of 0.6–1.25 mm. 2D T2w MRI was acquired with 0.43–0.72 mm in-plane resolution and a slice thickness of 3–4 mm. A slice gap of 0.5/1.2 mm was used for T2w MRI from two of the scanners.

### Network architecture

**Figure 1:**
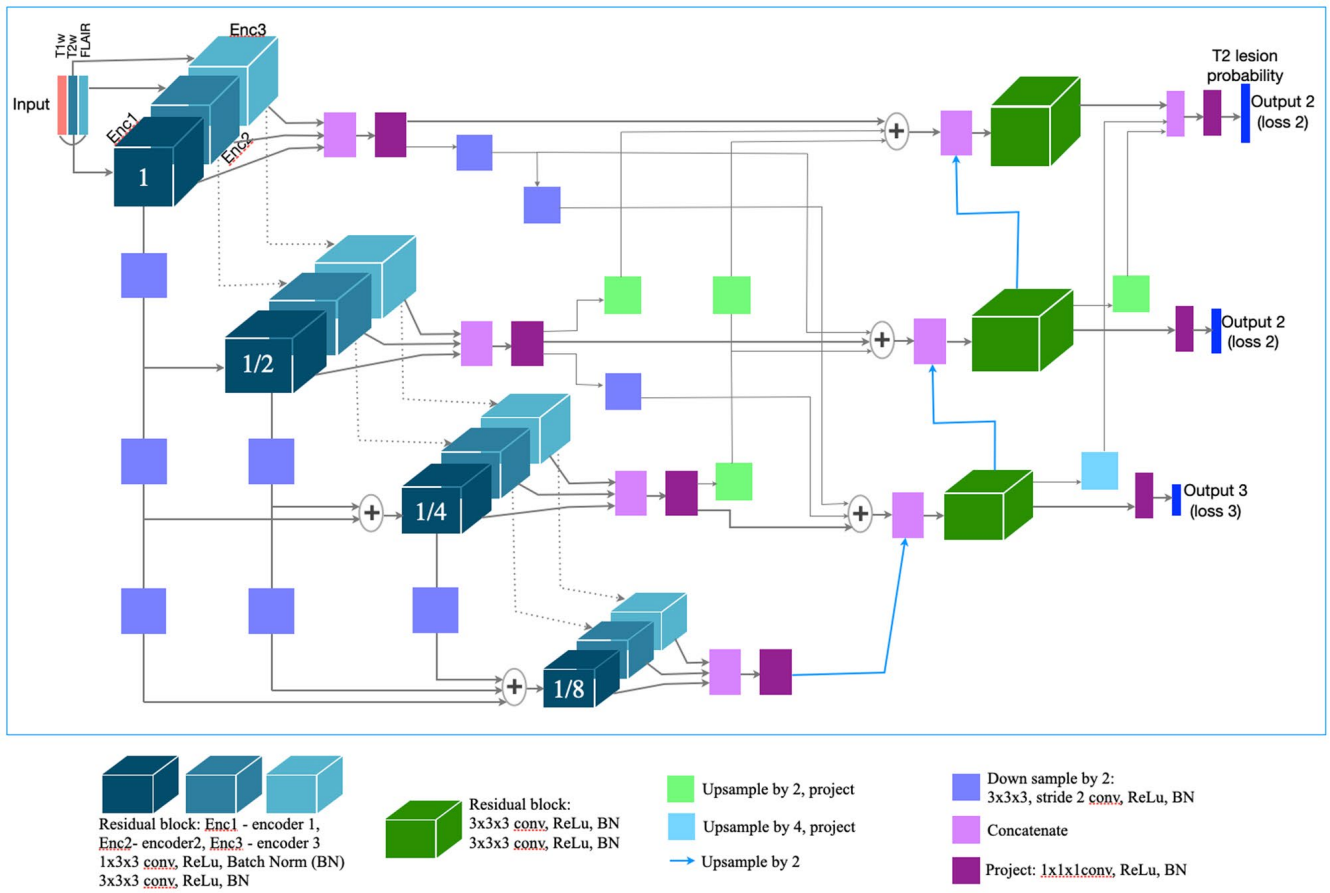
Network architecture of multi-arm U-Net for MS T2 lesion segmentation. The network uses three encoders: Encoders 2 and 3 capture the disjoint lesion information in FLAIR and T2w MRI, respectively; Encoder 1 captures the overlapping lesion information in T1p, T2w, and FLAIR MRI. The three encoders use dense input connectivity. The concatenation and projection of skip features at various levels/depths from the various encoders is shown. The decoder progressively up-samples the features from the main branch and concatenates with the skip features from the encoder. The network uses dense connectivity for skip features by down-sampling and up-sampling skip features from various levels as needed. The residual blocks used 1 × 3 × 3 and 3 × 3 × 3 convolutions, batch normalization (BN), and rectified linear unit (ReLu) activation layers. Down-sampling was performed using strided convolutions. To learn meaningful representation at various scales, the losses at various levels were combined and backpropagated during training. Enc, encoder; conv, convolution.

The network architecture is shown in Fig. 1. Though the segmentation and detection performance of the single-arm U-Net operating on 2.5D stacks of axial slices and 3D single-arm and multi-arm U-Nets operating on 3D patches were comparable in our previous analysis^20^, only the multi-arm U-Net with skip attention was able to identify treatment-related differences at the earliest follow-up of 24 weeks. Hence, we used a multi-arm U-Net with encoders extracting features separately from T2w and FLAIR sequences to better capture nonoverlapping T2 lesion information and jointly in the third encoder from all three MRI sequences (T2w, FLAIR, and T1p). As most MS lesions are small, we used a shallow network that was four levels deep and was designed to have equivalent receptive fields along the three orthogonal axes of the 3D input. Each encoder used residual blocks with 1 × 3 × 3 and 3 × 3 × 3 convolutions, batch normalization, and rectified linear unit activation layers. There is a loss of fine details when the extracted high-resolution features are downsampled to increase the receptive field. Use of dilated convolutions is memory intensive. Dense connectivity enables easier flow of information across scales and may improve the segmentation of thin and small structures. Hence, inspired by the parallel streams in HRNet^21^ for learning high-resolution representations, we customized our multi-arm U-Net to have a similar flow of features across various depths by using shortcut connections in the encoder, decoder, and skip features, while simultaneously replacing concatenation with summation of features to reduce memory requirements. The deeper encoding blocks received inputs from all the previous levels, where the features were down-sampled with strided convolutions to match the resolution at the current depth and then summed. The resulting features at each level from the three encoders were concatenated and projected using 1 × 1 × 1 convolution to reduce the number of extracted features to be used as skip features passed from the encoder to the decoder.

The skip features from the encoders were combined with features up-sampled using interpolation layers and refined progressively with residual blocks in a single decoder. The skip features at a given depth were obtained from the summation of the skip features at the various depths after down- or up-sampling to match the current resolution. The network predicted a lesion probability map at every depth and used dropout layers during training to reduce over-fitting.

### Model training and evaluation

The model was implemented in Python using Keras in Tensorflow^22^ and was trained using three-fold cross-validation with splits at the patient level. Input MRI data were rescaled to the same intensity range, clipped to remove intensity outliers, and normalized by z-scoring using the mean and standard deviation of intensities within the 1st and 99th percentiles. 3D patches of 32 × 96 × 96 from T2w, FLAIR, and T1p sequences concatenated along the channel dimension were used as inputs. The 3D patches were created using a sliding approach with overlaps (20%) and patches containing T2 lesions were retained in the training and validation sets. As the patches were relatively large, they included a large portion that did not contain any lesions and inclusion of negative patches decreased model performance. During training, half of the input images were augmented randomly on the fly with affine transformations and elastic deformations. The models were trained on NVIDIA V100 GPUs for 30 epochs using an Adam optimizer with an initial learning rate of 0.0001 and a batch size of 10. The training took 9 to 12 days.

The loss function was a combination of soft dice loss^23^ and weighted smooth truncated binary cross-entropy^24^. Smooth truncated loss was introduced to reduce the cross-entropy loss being dominated by few outliers having low predicted probabilities.

1 $$\begin{array}{c}L={\alpha L}_{FG-ST}+\beta {L}_{BG-ST}+{L}_{dice}\end{array}$$

2 $$\begin{array}{c}{{L}_{ST}=\left\{\begin{array}{c}-\mathrm{log}\left(\gamma \right)+ \frac{1}{2}\left(1-\frac{{p}_{i}^{2}}{{\gamma }^{2}}\right), {p}_{i}<\gamma \\ -\mathrm{log}\left(\gamma \right), {p}_{i} \ge \gamma \end{array}\right.; }L_{dice}= 1-\frac{2\sum_{i=1}^{n}{p}_{i}{q}_{i}}{\sum_{i=1}^{n}{p}_{i}^{2}+\sum_{i=1}^{n}{q}_{i}^{2}}\end{array}$$

L_dice_ is the soft dice loss; L_FG-ST_ is the smooth truncated cross-entropy loss of the foreground voxels; L_BG-ST_ is the smooth truncated cross-entropy loss of the background voxels; α and β are the weights for the foreground and background cross-entropy losses, respectively; p_i_ is the predicted probability of the ith voxel; q_i_ is the corresponding ground truth label; and γ is the minimum predicted probability below which the cross-entropy loss is approximated by a quadratic function. We varied γ from 0.02 to 0.15 over the epochs, α was varied as a sigmoid decay with lesion volumes and having a maximum weight of 100, and $\beta$ was varied as a sigmoid growth. These parameters were chosen empirically to reduce false positives without compromising sensitivity. The models were trained with deep supervision and the loss from the three levels were summed without any weighting.

Monte Carlo (MC) dropout was used during inference to get uncertainty estimates (*n* = 10 predictions). The predicted lesion probabilities of the three cross-validated models (3 × 10 predictions) were averaged to get the mean predicted lesion probability map, which was thresholded at 0.5 to get a binary lesion mask. Inference for a single visit was in the order of a few minutes (2–5 min). Generalization of the model trained on the MICCAI 2016 dataset was estimated on the consensus mask of the raters. The MRI inputs and consensus masks were resampled to the same resolution as the clinical trial datasets (axial slices; 1 × 1 × 3 mm^3^).

The mean dice coefficients (DCs), positive predictive values (PPVs), sensitivity or true positive rates (TPRs), and Pearson correlation coefficients of the predicted and GT volumes were used as metrics for evaluating segmentation performance^8^. Individual lesions were identified as connected components (with an 18-connectivity kernel) in the GT and predicted lesion masks. Lesions smaller than 3 voxels were excluded. A minimum overlap of 10% was used for detection. The lesion PPVs, lesion TPRs, lesion false positive rates (LFPRs), and Pearson correlation coefficients of the lesion counts predicted by the model and from GT masks were used as metrics for evaluating detection performance^8^.

### Model benchmarking

For benchmarking model performance, models with the architecture in Fig. 1 were trained on the preprocessed MRI from the ISBI 2015 train dataset and individual lesion masks from the two raters using four-fold cross-validation. The anisotropic convolutions in the residual blocks were replaced with isotropic convolutions to account for the isotropic voxel size. The performance score on the test set, which was a weighted combination of the segmentation and detection metrics, was obtained after uploading predicted masks to the challenge website (https://smart-stats-tools.org/lesion-challenge).

### Longitudinal changes in treatment and control arms

Percent change and absolute change of the T2 total lesion volume (TLV) from baseline to the end of trial period were secondary endpoints in the ORATORIO^13^ and OLYMPUS^14^ trials. The mean percent change in T2 TLV from baseline was estimated using GT and model-predicted lesion masks in the treatment and placebo arms of the ORATORIO and OLYMPUS trials. Analysis of covariance of baseline T2 TLV with geographical region (US versus rest of world) and age (≤ 45 versus > 45 years) as covariates was used to determine if the percent TLV change was statistically different between the treatment and placebo arms at the end of the trial period in the ORATORIO and OLYMPUS trials.

### Association of T2 lesion metrics with clinical outcome scores

The analysis relating baseline T2 lesion metrics to clinical outcome measures was performed in the OPERA II and ORATORIO trials. Baseline T2 TLV and total lesion count (TLC) were considered. Confirmed disability progression that was sustained for at least 24 weeks (CDP24) was defined as: a 20% or greater increase in the time to perform the timed 25-foot walk (T25FW24) or the nine-hole peg (9HPT24) tests; or an increase in expanded disability status score (EDSS) of 1.0 if baseline EDSS was ≤ 5.5, or 0.5 if baseline EDSS was > 5.5 were considered. Composite disability^13^ (CCDP24) was defined as the first confirmed occurrence of CDP24, T25FW24, and 9HPT24. Outcome events accumulated till the end of the study period were used.

Cox proportional hazards (CPH) models were used to determine the association of T2 lesion metrics with outcome measures. T2 TLV in mL was log-transformed. Kaplan–Meier (KM) curves for the T2 lesion metrics that showed at least a trend (*p* < 0.1) in the univariable CPH models were obtained by dichotomizing at the median value and were compared using a log-rank test. The analysis was performed in the two trials separately using T2 lesion metrics derived from GT lesion masks. Age, sex, baseline EDSS, treatment arm, and region were included as covariates in multivariable CPH models, along with the interaction of the treatment arm with T2 lesion measure. The analysis was repeated with T2 lesion metrics estimated from the multi-arm model predictions. Statistical analysis was performed in R (R version 4.0.1, R Foundation for Statistical Computing) and *p* < 0.05 was considered indicative of a statistically significant difference.

## Results

The clinical and GT lesion characteristics of the patients in these datasets are summarized in Table 1.

**Table 1:** Summary of clinical and GT lesion characteristics of OCR and RXB clinical trial datasets in patients with MS.

Metric	OPERA I	OPERA II	ORATORIO	OLYMPUS
Clinical characteristics (n)				
Number of patients	796	798	714	416
Number of visits	2941	2839	2605	1787
Gender	M: 272, F: 524	M: 275, F: 523	M: 361, F: 353	M: 210, F: 206
Age (years)				
Mean ± SD	37.15 ± 9.3	37.41 ± 8.97	44.53 ± 8.02	49.95 ± 8.9
Range	18–56	18–55	18–56	18–66
Treatment arms (n)	OCR, INF	OCR, INF	OCR, placebo	RXB, placebo
Baseline	397, 399	398, 400	476, 238	276, 140
W6	–	–	–	266, 133
W24	378, 365	369, 357	451, 224	–
W48	371, 349	357, 320	440, 212	234, 119
W96	354, 328	341, 297	–	213, 112
W120	–	–	392, 172	*W122*: 196, 98
Baseline GT TLV (mL)				
Mean ± SD	9.84 ± 12.44	9.96 ± 12.8	11.79 ± 14.09	13.81 ± 15.42
Range	0.017–86.52	0.00–95.85	0.00–97.27	0.05–129.99

*GT* ground truth, *INF* interferon, *OCR* ocrelizumab, *RXB* rituximab, *SD* standard deviation, *TLV* total lesion volume, *W* week.

### T2 lesion segmentation and detection performance

The segmentation and detection performance of the multi-arm U-Net on the internal and external test sets are provided in Table 2. Among the internal test sets, the model performed similarly on the OPERA II and ORATORIO datasets (mean DCs of 0.72 and 0.7 and mean lesion true positive rate [LTPR] of 0.84 and 0.87, respectively), but had a marginally reduced performance for the OLYMPUS dataset (mean DC of 0.66 and mean LTPR of 0.72). Specifically, the sensitivity for segmentation (TPR of 0.79, 0.82 versus 0.67) and lesion detection (LTPR of 0.84, 0.87 versus 0.72) were reduced, and the precision (LPPV of 0.88, 0.86 versus 0.86) and false positive rate (LFPR of 0.18, 0.15 versus 0.18) for lesion detection were equivalent to the other two trial datasets. On the MICCAI 2016 dataset, the model achieved a mean DC of 0.623 and a mean lesion detection sensitivity of 0.681 against the consensus masks, while the LFPR of 0.25 was higher when compared with the internal test sets. The average DCs between the human raters varied from 0.565 to 0.677, and the U-Net had a mean dice score of 0.539 against the individual raters (Supplementary Table S1).

**Table 2:** Segmentation and lesion detection performance of the multi-arm U-Net on internal and external test sets.

Segmentation	Test set	PPV	TPR	DC	r_vol_
Internal	OPERA II	0.695 ± 0.148	0.785 ± 0.146	0.72 ± 0.12	0.969
	ORATORIO	0.64 ± 0.161	0.818 ± 0.128	0.698 ± 0.124	0.962
	OLYMPUS	0.676 ± 0.152	0.672 ± 0.133	0.66 ± 0.123	0.975
External	MICCAI 2016*	0.736 ± 0.18	0.562 ± 0.151	0.623 ± 0.15	0.975

Detection	Test set	LPPV	LTPR	LFPR	r_count_
Internal	OPERA II	0.884 ± 0.136	0.837 ± 0.134	0.178 ± 0.138	0.958
	ORATORIO	0.859 ± 0.153	0.866 ± 0.117	0.152 ± 0.128	0.949
	OLYMPUS	0.859 ± 0.143	0.717 ± 0.142	0.175 ± 0.128	0.898
External	MICCAI 2016*	0.683 ± 0.195	0.681 ± 0.177	0.252 ± 0.174	0.942

Means ± standard deviations reported. *r*_*vol*_ Pearson correlation coefficient of predicted and ground truth (GT) total lesion volumes, *r*_*count*_ Pearson correlation coefficient of predicted and ground truth total lesion counts.

*Performance metrics were estimated using the consensus mask as the GT.

**Figure 2:**
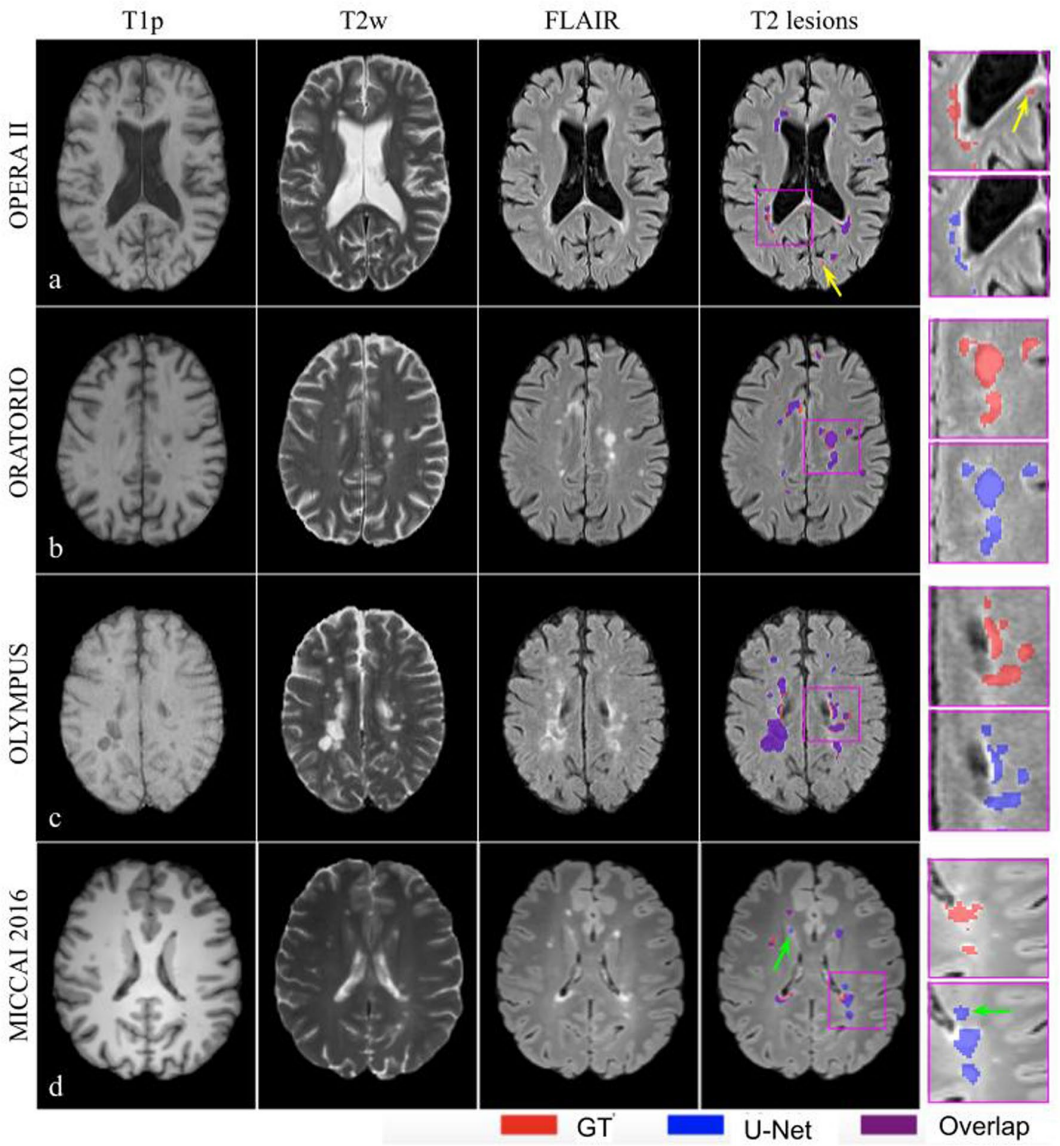
Illustrative examples of T2 lesion segmentations from GT and multi-arm U-Net predicted masks. The inputs to the model: T1p, T2w, and FLAIR MRI, are shown in columns 1–3. Column 4 shows the overlay of T2 lesions on FLAIR MRI, with GT shown in red, model predictions in blue, and their overlap shown in purple. Magnified insets are shown in column 5 for a better visualization of the segmentation agreement between model predictions and GT annotations. Yellow arrows point to false negative lesions and green arrows point to false positive lesions. Rows (**a**–**c**) are examples of T2 lesion segmentations from internal test sets of a patient with relapsing MS in the OPERA II trial (**a**) and patients with PPMS in the ORATORIO (**b**) and OLYMPUS (**c**) trials. Row d corresponds to an illustrative patient with MS in the MICCAI 2016 dataset used as the external test set here. The examples highlight the good agreement of model predictions with GT masks and have high sensitivity to detect a majority of small lesions across various test sets. *GT* ground truth.

Representative examples of T2 lesion segmentations by the multi-arm U-Net are shown in Fig. 2. Visually, there is generally a good agreement between the model-predicted and GT masks, and the model had a high sensitivity to detect a majority of the small lesions in these patient datasets. The false positive and false negative lesions tended to be smaller. Consistent with the qualitative results, the good agreement of our model with GT annotations is confirmed with the high correlation coefficients for TLVs (*r* > 0.96) and TLCs (*r* ≥ 0.9). For a better understanding of the segmentations for the various lesion loads, the scatter and Bland–Altman plots for TLVs are provided in Supplementary Fig. S1. From the Bland–Altman plots, we observed that the model tended to underestimate higher lesion loads in the OLYMPUS and MICCAI 2016 datasets.

**Figure 3:**
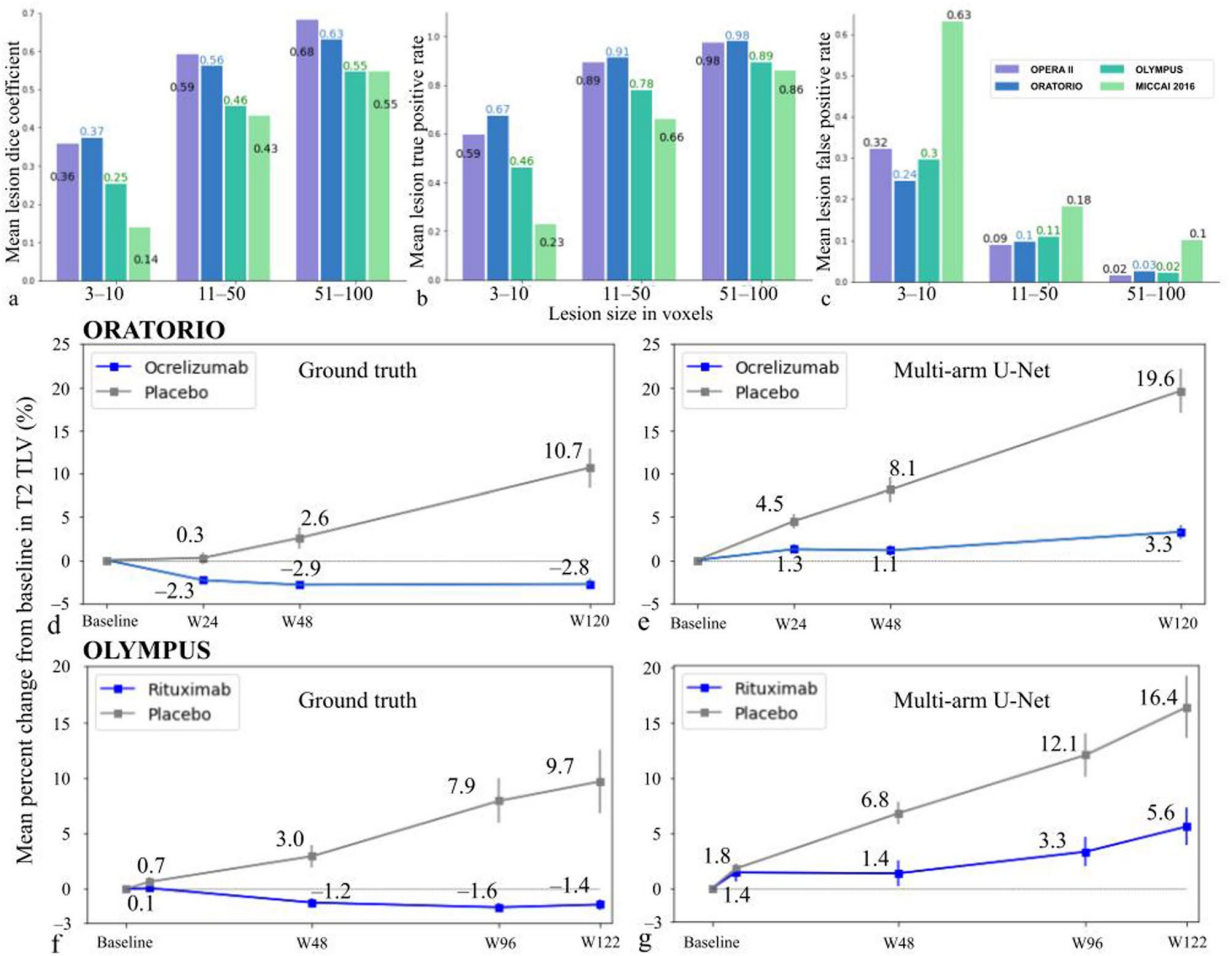
T2 lesion detection performance for different lesion size groups and reproduction of secondary imaging endpoint on T2 TLV. Bar plots of mean lesion DC (**a**), mean lesion true positive rate (**b**) and mean lesion false positive rate (**c**) of T2 lesion detection of the proposed multi-arm U-Net for the three smaller lesion size groups. The performance was similar in OPERA II and ORATORIO trials, whilst it was slightly reduced for the OLYMPUS trial and further reduced for the MICCAI 2016 dataset, which could be attributed to differences in MRI acquisition and annotation styles. Across the various datasets, lesion detection performance improved with increase in lesion size. Percent change in T2 TLV from baseline to Week 120 was a secondary imaging endpoint in the ORATORIO trial. This endpoint was reproduced by using either GT masks (**d**) and multi-arm U-Net predicted masks (**e**) in the ORATORIO trial, showing similar separation between the ocrelizumab and placebo arms. Longitudinal trends from a similar analysis of the OLYMPUS trial using GT masks (**f**) and predicted masks (**g**) exhibited similar relative separation between the rituximab and placebo arms. The shift in the mean values may be due to the false positives and false negatives in the model predictions. DC, dice coefficient; GT, ground truth; TLV, total lesion volume; w, week.

The individual lesion size distribution was skewed more towards smaller sizes. In the OPERA II dataset of patients with RMS, 41% (*n* = 55,192) and 83% (*n* = 111,964) of lesions had maximum sizes of 10 voxels and 50 voxels, respectively. The distributions were analogous in the ORATORIO and OLYMPUS datasets of patients with PPMS, with 43% (*n* = 54,709) and 84% (*n* = 107,602); and 46% (*n* = 42,479) and 84% (*n* = 77,416) of lesions having maximum sizes of 10 voxels and 50 voxels, respectively. In the MICCAI 2016 dataset, the lesion size distribution was still skewed towards small lesions and had 23% (*n* = 406) and 78% (*n* = 1344) of lesions in the same size groups. In the three small lesion size groups, mean lesion DC (Fig. 3a), LTPR (Fig. 3b), and LFPR (Fig. 3c) improved as the size of the lesions increased in the various datasets. The model predictions had a lower DC and higher LFPR in the MICCAI 2016 dataset, distinctly for the smallest size group of 3–10 voxels. In the MICCAI 2016 dataset, compared with Raters 3 and 5, who had the least and best agreements with the consensus masks, respectively, the LFPR of the model was comparable to that of Rater 3 (0.63 versus 0.5) for the smallest size group of 3–10 voxels and better than Rater 3 for lesions with sizes of 11–50 voxels (0.18 versus 0.3). However, lesion DC and LTPR were noticeably lower than both raters for lesions with sizes ≤ 50 voxels (Supplementary Fig. S2). The multi-arm U-Net had 30% fewer parameters (5.9 M) than a 3D U-Net (7.7 M) with the same number of filters at the various depths (Supplementary Table S2). In the ablation experiments, the mean performance of the different models varied marginally but there was an improvement of LFPR for the smallest lesion group (3–10 voxels) with the addition of shortcut connections in the single-arm U-Net (Supplementary Table S2).

The multi-arm U-Net achieved an overall score of 93.039 in the ISBI 2015 challenge dataset, which is 0.319 points lower than the top-performing model (Table 3). The model ranked within the top 50 of over 1500 submissions to the challenge website. It had one of the lowest absolute volume differences, and the DC, PPV, LTPR, and LFPR were comparable to other best performing models.

**Table 3:** Performance of the multi-arm U-Net on the test set of the ISBI 2015 challenge.

Team	Score	DC	PPV	LTPR	LFPR	AVD
Best model	93.36	0.669	0.886	**0.538**	0.125	0.396
Zhang et al.^2^	93.21	0.643	0.908	0.520	0.124	0.428
Ours (5.9 M)	93.04	0.677	0.865	0.520	0.143	**0.362**
Brugnara et al.^5^* (19.1 M)	93.03	**0.681**	0.855	0.538	0.157	0.366
Hashemi et al.^28^	92.48	0.584	**0.921**	0.414	**0.087**	0.497
Aslani et al.^3^	92.12	0.611	0.899	0.410	0.139	0.454

The highest or lowest values are in bold.

*3D nnU-Net trained for MS T2 lesion segmentation. The number of model parameters for multi-arm and nnU-Net is provided within parenthesis.

*AVD* absolute volume difference, *DC* dice coefficient, *LFPR* lesion false positive rate, *LTPR* lesion true positive rate, *PPV* positive predictive values.

### Longitudinal changes in treatment and control arms

Ocrelizumab treatment response estimated as the reduced percent increase in T2 TLV from baseline in the treatment arm when compared with the placebo arm was used to ascertain the model’s ability to capture longitudinal trends. In the ORATORIO dataset, analysis of the ground truth masks found that the mean percent change in T2 TLV showed a marked reduction from baseline to Week 24 and continued to remain stable at less than a 3% decrease through Week 120 in the ocrelizumab arm. There was no such reduction in the placebo arm, with the T2 TLV remaining stable till Week 24 and then increasing consistently to approximately 11% through Week 120 (Fig. 3d). For the model predicted masks, both treatment and placebo arms exhibited an increase in percent change in T2 TLV, with the treatment arm having a reduced rate of increase compared with the placebo arm (Fig. 3e). Though the mean values were different, the separations of the arms when using the GT masks were 2.6%, 5.5%, and 13.5% at Weeks 24, 48, and 120, respectively, which were similar to 3.2%, 7%, and 16.3% when using model predictions. The percent TLV change was statistically different between the ocrelizumab and placebo arms at Week 120 in the analysis of covariance (*p* < 0.001) for both the ground truth and predicted masks. The longitudinal trend of the different model variants in the ablation experiments (Supplementary Fig. S3) showed similar separation between the treatment and control arms and the single-arm model with shortcut connections in the encoder, skip features, and decoder had the least difference with the separations estimated from the manual annotations.

The analysis of the OLYMPUS dataset revealed a similar trend in the percent change of T2 TLV over the trial (Fig. 3f,g). However, GT masks produced slightly lower slopes for both arms (Fig. 3f) when compared to the ORATORIO trial (Fig. 3d), while the predicted slopes were slightly lower for the treatment arm and slightly higher for the placebo arm (Fig. 3g). The separations of the arms were 0.6%, 4.2%, 9.5%, and 11.1% at Weeks 6, 48, 96, and 122 when using the GT masks, and 1.4%, 5.4%, 8.8%, and 10.8% when using model-predicted masks. The percent TLV changes at Week 122 were statistically different in the analysis of covariance (*p* < 0.001) for both the ground truth and predicted masks.

### Association of T2 lesion metrics with clinical outcome scores

**Figure 4:**
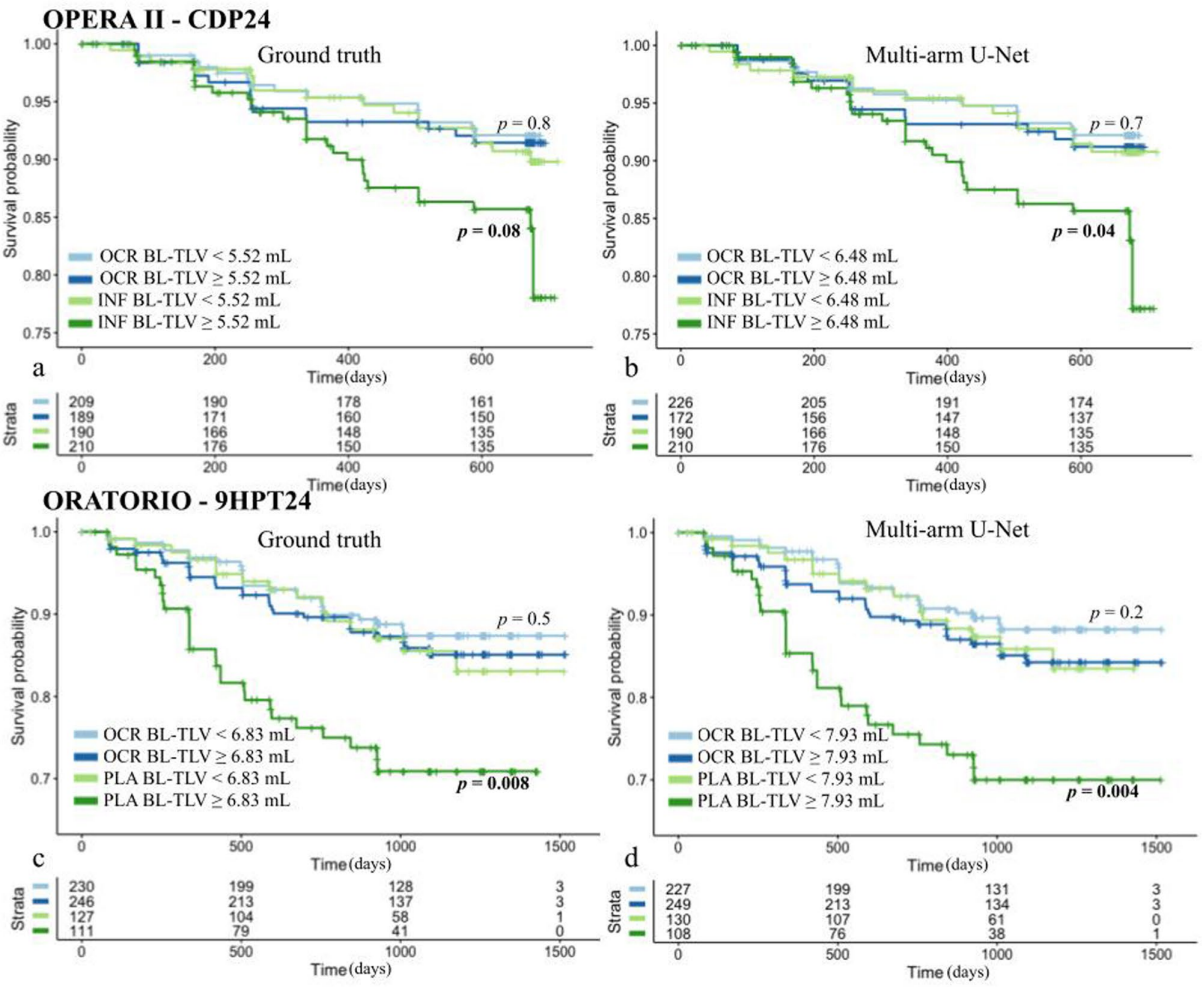
Association of baseline T2 TLV with confirmed disability progression sustained for at least 24 weeks in MS clinical trials. KM curves of CDP24 dichotomized by median T2 TLV at baseline from GT masks (**a**) and predicted masks (**b**) showed a good separation in the interferon arm, but not in the ocrelizumab arm, of the OPERA II trial. KM curves of 9HPT24 dichotomized by median baseline T2 TLV revealed a clear separation in the placebo arm when using GT masks (**c**) and predicted masks (**d**), but not in the Ocrelizumab arm of the ORATORIO trial. The median values were estimated over the whole cohort and applied to the treatment and control arms separately. *P* values are from a log-rank test to compare the KM curves. *9HPT24* nine-hole peg test (sustained for at least 24 weeks), *BL* baseline, *CDP24* confirmed disability progression (sustained for at least 24 weeks), *GT* ground truth, *IFN* interferon beta-1-a, *KM* Kaplan–Meier, *OCR* ocrelizumab, *PLA* placebo, *TLV* total lesion volume.

In the OPERA II dataset, log-transformed baseline T2 TLV showed a trend (*p* = 0.06) towards association with CDP24 when using the GT or model-predicted masks (Table 4) in the univariable CPH analysis. The KM curves stratified by median baseline T2 TLV showed a separation in the control arm but not in the treatment arm (Fig. 4a). The separation of KM curves in the control arm was statistically significant in a log-rank test when using model-predicted masks (Fig. 4b). In the multivariable CPH model, after accounting for covariates, the log-transformed baseline T2 TLV had a statistically signification association with CDP24 (Table 4). Baseline T2 TLC was not associated with the four outcome measures. In the univariable CPH analysis of the ORATORIO dataset, log-transformed baseline T2 TLV was associated with 9HPT24 but was not significant in a multivariable CPH model (Table 4). KM curves from GT and model-predicted masks revealed a clear separation in the placebo arm and no separation in the ocrelizumab arm (Fig. 4c,d). Baseline T2 TLC from GT masks was associated with 9HPT24 (*p* = 0.016; HR 1.006; 95% CI 1.001–1.011) in the univariable, but not in the multivariable, CPH model.

**Table 4:** Association of baseline T2 TLV with confirmed disability progression sustained for 24 weeks in the OPERA II and ORATORIO trials.

Trial	Analysis and variable	CPH model with GT BL T2 TLV		CPH model with predicted BL T2 TLV	
		Hazard ratio*	*p* value	Hazard ratio*	*p* value
OPERA II (CDP24: 74 events)	***Univariable:*** BL T2 TLV	1.18 (0.995, 1.4)	***0.057***	1.19 (0.998, 1.425)	***0.053***
	***Multivariable***				
	Arm: Ocr	1.37 (0.618, 3.045)	0.437	1.38 (0.586, 3.228)	0.465
	Gender: M	1.00 (0.618, 1.625)	0.994	1.00 (0.618, 1.628)	0.99
	Region: US	1.63 (1.003, 2.657)	**0.048**	1.63 (1.001, 2.65)	**0.049**
	BL EDSS: < 4	1.29 (0.732, 2.267)	0.379	1.29 (0.731, 2.266)	0.382
	Age	1.01 (0.986, 1.04)	0.354	1.01 (0.986, 1.04)	0.352
	BL T2 TLV	1.49 (1.153, 1.926)	**0.002**	1.48 (1.139, 1.924)	**0.003**
	Arm Ocr: BL T2 TLV	0.65 (0.459, 0.927)	**0.017**	0.67 (0.466, 0.966)	**0.032**
ORATORIO (9HPT24: 101 events)	***Univariable:*** Bl T2 TLV	1.22 (1.047, 1.411)	**0.010**	1.23 (1.048, 1.452)	**0.012**
	***Multivariable***				
	Arm: Placebo	1.29 (0.627, 2.678)	0.484	1.12 (0.485, 2.58)	0.793
	Gender: M	1.54 (1.029, 2.295)	**0.036**	1.52 (1.02, 2.275)	**0.04**
	Region: US	1.36 (0.803, 2.311)	0.252	1.34 (0.791, 2.278)	0.275
	BL EDSS: < 4	0.44 (0.263, 0.743)	**0.002**	0.44 (0.261, 0.736)	**0.002**
	Age	0.97 (0.951, 0.997)	**0.026**	0.97 (0.951, 0.997)	**0.025**
	BL T2 TLV	1.10 (0.914, 1.327)	0.312	1.09 (0.891, 1.337)	0.399
	Arm Placebo: BL T2 TLV	1.18 (0.875, 1.587)	0.280	1.24 (0.895, 1.716)	0.196

Significant values are in bold and bolditalics.

Region: US versus rest of world; BL EDSS: < 4 versus ≥ 4; log-transformed TLV was used in the CPH models.

*Numbers in parenthesis are 95% confidence intervals.

## Discussion

The multi-arm U-Net developed in this study had good segmentation performance (mean DC ≥ 0.623) and high lesion detection sensitivity (LTPR ≥ 0.681), with an acceptable false positive rate (LFPR ≤ 0.252), when evaluated on other large clinical trial datasets and an external dataset acquired with a different MRI protocol. The model predictions showed a similar separation between the treatment and placebo arms for percent change in T2 TLV when compared with the manual annotations (*p* < 0.001) for both the ORATORIO and OLYMPUS trials. Baseline T2 TLVs from both GT and model-predicted masks revealed analogous association with clinical outcomes and a good separation in the KM curves for the control arms in the OPERA II and ORATORIO trials.

MRI datasets from phase 3 clinical trials are typically large but are acquired with a standardized MRI acquisition protocol. In comparison to a 2D U-Net^11^ developed for joint MS T2 lesion and anatomical brain segmentation on a baseline MS clinical trial dataset with multimodal inputs of PDw, T2w, FLAIR, and T1w MRI, the multi-arm U-Net had a lower mean LFPR of less than 0.18 across the three clinical trial datasets against a LFPR of 0.34 of the 2D U-Net. The mean DC and lesion detection sensitivity (LTPR) were comparable with the 2D U-Net, which achieved a mean DC of 0.82 and a mean LTPR of 0.79, with the multi-arm U-Net achieving a mean DC greater than 0.65 and a mean LTPR greater than 0.7. This 2D U-Net, when trained on a multimodal MRI input of T1w, T2w, and FLAIR MRI^25^, achieved a mean DC of 0.73, a LTPR of 0.85, and a LFPR 0.58, which was higher than 0.18 from our model. The lesion detection performance was reduced for smaller lesions (≤ 30 μL), with the multi-arm model akin to the reduction (< 20 μL) noticed in these models^11,25^. Among the internal test sets, the performance was reduced for the OLYMPUS trial, which might indicate reduced model generalization to datasets acquired with a slightly different MRI protocol.

Models trained on RWD often have smaller training and validation sets, but have better representation of the variation in MRI acquisition protocols. A 2D, multi-arm U-Net^3^ trained on a small single institutional dataset (*n* = 37) had a reduced LTPR of 0.45, as opposed to greater than 0.7, and an improved LFPR of 0.08, as opposed to less than 0.18 from our 3D, multi-arm U-Net with dense connectivity. 3D nnU-Net trained and evaluated on a single center dataset (*n* = 334) for joint MS T2 and T1 contrast-enhancing lesion segmentation attained a higher DC of 0.85 and had reduced detection performance in the follow-up scans, with a 5.5% reduction in LTPR when compared with baseline. Similarly, there was a 2.3% to 5.5% drop in the DC and a 1% to 4% drop in the LTPR from our model for the follow-up scans when compared with baseline scans, which could have been driven by the reduced lesion load of patients undergoing aggressive therapies. The mean lesion DC from a combination approach^26^ using unsupervised machine learning and attention U-Net^27^ trained on a multicenter RWD (*n* = 159) of T1w and FLAIR MRI was 0.64, which was higher than 0.62 seen in the MICCAI 2016 dataset, the same as that seen for OLYMPUS, and lower than 0.68 and 0.72 seen in the ORATORIO and OPERA II test sets, respectively. However, as these models were trained and tested on different datasets, a direct comparison of their performances remains unfavorable. Hence, the next set of models were evaluated on the MICCAI 2016 dataset.

Joint segmentation of MS T2 lesions and brain anatomical regions improved T2 lesion segmentation performance^7^ and model generalization with varying MRI contrast^6^. The 2.5D DeepSCAN model had a considerably lower number of parameters (< 0.5 M) than the multi-arm U-Net. The 3D nnU-Net and 2.5D DeepSCAN models trained on RWD (*n* = 122) from a single center and evaluated against the consensus masks of the training set of the MICCAI 2016 (*n* = 15) dataset^7^ achieved mean DCs of 0.61 and 0.66 for nnU-Net and DeepSCAN, respectively, when trained to segment T2 lesions and brain regions jointly, and mean DCs of 0.62 and 0.6, respectively, when trained to segment T2 lesions only. Correspondingly, our model trained on the OPERA I trial dataset and evaluated against the consensus masks of the MICCAI 2016 (*n* = 53; train and test sets) dataset had a mean DC of 0.62, and a mean DC of 0.54 (Supplementary Table S1) when evaluated against individual raters. The mean DC against the seven individual raters was 0.53 and 0.59 for joint segmentation; 0.54 and 0.53 for segmenting T2 lesions only with nnU-Net and DeepSCAN models and 0.57 with the generative model^6^. Of note, the MICCAI 2016 dataset was acquired with a standardized MRI protocol, and it was down-sampled to match the resolution of the clinical trial datasets in our analysis. Therefore, a reduction in the agreement among the various raters and model performance metrics was expected. Nonetheless, our model generalized reasonably well to the external dataset when compared with the consensus masks and individual raters. The decrease in performance was more noticeable for the smallest lesion size group (3–10 voxels; Fig. 3a–c). Despite the LFPR being high for smaller lesion sizes, it was comparable to that of Rater 3, but the mean DC and LTPR of the model were lower. This could be attributed to a combination of data shift (change in distribution of intensities and noise characteristics) and a probable difference in manual lesion annotation styles. Another aspect is that multimodal MRI in RWD are frequently resampled to an isotropic, 1 mm^3^ resolution, to account for the different resolutions and to better utilize the high-resolution MRI for manual GT annotation when available. The multi-arm U-Net developed in this study was trained on the native resolution of the clinical trial datasets with a 3 mm slice thickness for computational efficiency. Training and evaluation on up-sampled, isotropic, 1 mm^3^ MRI datasets may improve the performance.

Benchmarking of model performance on the ISBI 2015 challenge dataset, where the models were trained with the same training set and evaluated on the same test set, placed this model among the top 50 performers and revealed an identical overall test score as the nnU-Net^5^, but lower than the 2.5D Tiramisu model^2^. The multi-arm U-Net (5.9 M) was 3X smaller than 3D full resolution nnU-Net^30^ (19.1 M). We attribute the high performance to various components of the multi-arm U-Net, namely: separate encoders for better extraction of nonoverlapping lesion features from T2w and FLAIR MRI; dense input and skip connectivity for better propagation of features; a combined loss function with suppression of large errors from a few outliers; and MC dropout during inference to reduce false positives and uncertainty estimation.

While most of T2 lesion segmentation models focus on cross-sectional segmentation and lesion detection performance, Brugnara et al*.* looked into the longitudinal trends in the T2 lesion volumes of patients qualitatively^5^. The multi-arm model described here had a sufficiently high sensitivity to reproduce the secondary imaging endpoint on percent change of T2 TLV with reference to baseline and maintain the relative separation of the treatment and controls arms in the ORATORIO and OLYMPUS trials in patients with PPMS. The difference in the mean values was probably driven by the false positives and false negatives in the model predictions. As the OPERA trials of patients with RMS had lower lesion loads, the percent changes in T2 TLVs were comparatively less stable. In addition to the longitudinal trends, the model-predicted and GT masks had good cross-sectional performances at baseline, seen by the similar associations with outcome clinical scores in the univariable and multivariable CPH models and separation in the KM curves.

A minimum lesion size threshold of 3 voxels is commonly used in MS irrespective of the voxel resolution. The higher prevalence of small lesions poses unique challenges in balancing sensitivity and amplification of false positives. Weighting the binary cross-entropy loss from the lesion voxels heavily in comparison to the nonlesion voxels helps to address the class imbalance. However, using a large constant weight amplifies false positives and weights proportional to inverse of lesion volumes have been used. We also experimented with asymmetric^28^ and Tversky^29^ loss functions in our earlier models but the performance of the trained models was not satisfactory. The sigmoid weighting of the cross-entropy loss provided a good balance of the number of false positives and sensitivity but resulted in nonlinear segmentation errors.

Besides cross-sectional assessment of T2 lesions, there is an active interest in quantifying new and enlarging lesion activity longitudinally. In this regard, as part of the MSSEG2 challenge various approaches were developed for segmenting new T2 MS lesions^31^. However, reliable detection of subtle and small changes in lesions is challenging. Longitudinal tracking of lesions from cross-sectional lesion segmentations may be more stable, especially when the MRI acquisitions for the two visits differ appreciably. Hence, we focused on T2 lesion segmentation here and the segmentation of new and enlarging T2 lesions will be explored further in a future study.

Our study had some limitations. Though the clinical trial datasets were acquired across multiple sites and from a variety of scanners, the MRI acquisition protocol was standardized and does not reflect the same degree of variation seen in RWD. We only had single reads from a group of experts for use as GT annotations. The infratentorial lesions were not annotated consistently and the MRI sequences were not ideal for detecting cortical lesions reliably.

In conclusion, we have developed a 3D, multi-arm U-Net for MS T2 lesion segmentation trained with a large, phase 3, multicenter clinical trial dataset of patients with RMS. The model generalized well to other clinical trial datasets of patients with RMS and PPMS, and to an external test set, and also highlighted the areas where the performance is mismatched. The developed model produced comparable separation between treatment arms for the T2 lesion volume endpoint and exhibited similar association of baseline T2 lesion volumes with clinical disability scores compared with manual lesion annotations. Our method has the potential to provide a good initialization of lesion annotations for manual verification and correction; and thereby, reduce rater burden for lesion characterization in clinical trials of patients with MS; however, its application to routine clinical care will require the model to be fine-tuned on large, heterogenous cohorts, to capture the wider disease pathology and differences in MRI acquisition.

## Supplementary Information

Supplementary Information.

## Data Availability

Qualified researchers may request access to individual, patient-level data through the clinical study data request platform (https://clinicalstudydatarequest.com). Further details on Roche’s criteria for eligible studies are available at https://clinicalstudydatarequest.com/Study-Sponsors/Study-Sponsors-Roche.aspx. For further details on Roche’s Global Policy on the Sharing of Clinical Information and how to request access to related clinical study documents, see https://www.roche.com/research_and_development/who_we_are_how_we_work/clinical_trials/our_commitment_to_data_sharing.htm.
