# Supplementary material for: Multi-arm U-Net with dense input and skip connectivity for T2 lesion segmentation in clinical trials of multiple sclerosis
Source: Sci Rep. 2023 Mar 13;13:4102. doi: 10.1038/s41598-023-31207-5 (PMC10011580; doi:10.1038/s41598-023-31207-5)
Supplement: Supplementary file 1 — Supplementary Information. [file 41598_2023_31207_MOESM1_ESM.pdf]

Table S1. Average dice coefficients corresponding to lesion segmentation agreement among the seven raters and the multi-arm U-Net predictions in the resampled MICCAI 2016 dataset

	R1	R2	R3	R4	R5	R6	R7	Ours
R1	–	0.697	0.562	0.756	0.781	0.565	0.559	0.521
R2	0.697	–	0.541	0.703	0.699	0.532	0.522	0.493
R3	0.562	0.541	–	0.558	0.571	0.559	0.597	0.538
R4	0.756	0.703	0.558	–	0.852	0.579	0.556	0.533
R5	0.781	0.699	0.571	0.852	–	0.587	0.571	0.538
R6	0.565	0.532	0.559	0.579	0.587	–	0.604	0.589
R7	0.559	0.522	0.597	0.556	0.571	0.604	–	0.561
Mean	0.653	0.616	0.565	0.668	0.677	0.571	0.568	0.539

Abbreviation: R1–7, individual raters.

Figure S1. Correlation and Bland–Altman plots of TLVs predicted by multi-arm U-Net

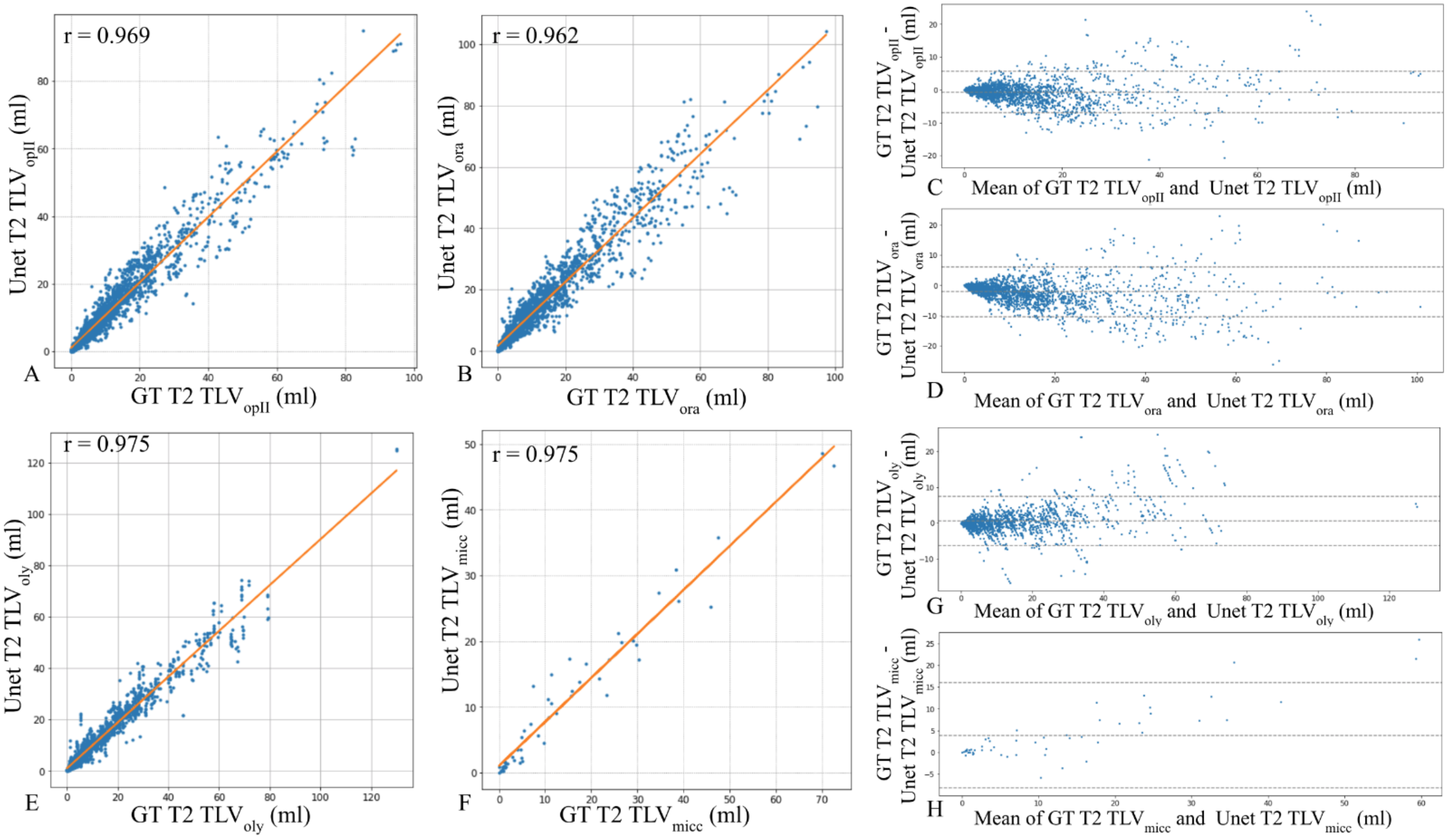

Correlation plots of T2 TLV in the internal (a, b, and e) and external test sets (f) showed good agreement between the GT masks and model predictions. Associated Bland–Altman plots are provided in (c, d, g, and h). From the Bland–Altman plots, the U-Net exhibited a trend towards underestimation, with an increase in TLV in the OLYMPUS trial (g) and MICCAI 2016 (h) dataset. Orange lines are lines of best fit for the points. GT, ground truth; micc, MICCAI; oly, OLYMPUS; opII, OPERA II; ora, ORATORIO; TLV, total lesion volume.

Figure S2. T2 lesion detection performance for different lesion size groups in the MICCAI 2016 dataset

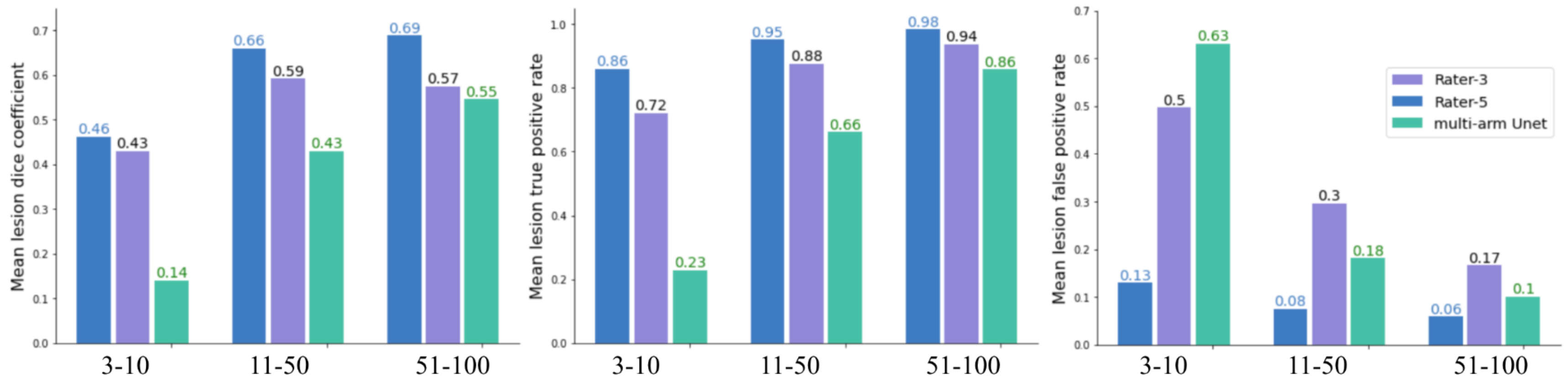

Bar plots of mean lesion dice coefficients (a), mean lesion true positive rates (b) and mean lesion false positive rates (c) of T2 lesion detection of Raters 3 and 5, who had the least and best agreement with the consensus masks and the proposed multi-arm U-Net for the three smaller lesion size groups, respectively. The mean lesion dice coefficient and lesion true positive rates of the multi-arm U-Net were noticeably lower than both raters for lesions with a size of 3–50 voxels, and the mean lesion false positive rate was comparable to that of Rater 3.

Ablation studies

The ablation studies to understand the contribution of the different architectural choices were performed on one of the cross-validation folds. The models were trained for 25 epochs using the baseline MRI of OPERA I dataset and tested on ORATORIO dataset. We trained five model variants with increasing levels of complexity and the number of filters in the single-arm U-Net were distributed equally to the three arms in the multi-arm U-Net. The multi-arm model was approximately 30% smaller than the 3D U-Net (7.7M) and had half the number of parameters (5.9M) compared with 3D U-Net with shortcut connections in the encoder, decoder, and skip features (13.1M, Table S2). The multi-arm U-Net developed here is comparable to using grouped convolutions, leading to the reduction in model size. The mean segmentation and detection performance of the model variants varied marginally; lesion detection sensitivity improved slightly with the addition of shortcut connections in the encoder but this also increased the false positives; adding shortcut connections for the skip features reduced the false positives but lead to reduction of lesion detection sensitivity. Addition of skip connections in the decoder increased the number of false positives when compared with base U-Net. The multi-arm U-Net achieved comparable performance as the single-arm U-Nets with a considerably smaller number of parameters (Table S2).

Table S2. Model size and performance of the different variants with increasing levels of complexity

Models	#parameters	DC	TPR	LTPR	LFPR
3D U-Net	7.662M	0.686 ± 0.128	0.817 ± 0.126	0.868 ± 0.116	0.181 ± 0.141
U-Net + enc sc	12.481M	0.681 ± 0.131	0.830 ± 0.115	0.886 ± 0.105	0.267 ± 0.198
U-Net + enc + skip sc	13.065M	0.688 ± 0.128	0.812 ± 0.125	0.865 ± 0.120	0.177 ± 0.139
U-Net + enc + skip + dec sc	13.071M	0.686 ± 0.129	0.814 ± 0.125	0.868 ± 0.119	0.239 ± 0.176
Multi-arm U-Net	5.914M	0.685 ± 0.128	0.819 ± 0.124	0.872 ± 0.115	0.183 ± 0.140

Abbreviations: DC, dice coefficient; dec, decoder; enc, encoder; LFPR, lesion false positive rate; LTPR, lesion true positive rate; sc, shortcut connections; TPR, true positive rate.

Figure S3. Reproducing the secondary imaging endpoint on T2 total lesion volume with the different model variants in the ORATORIO dataset

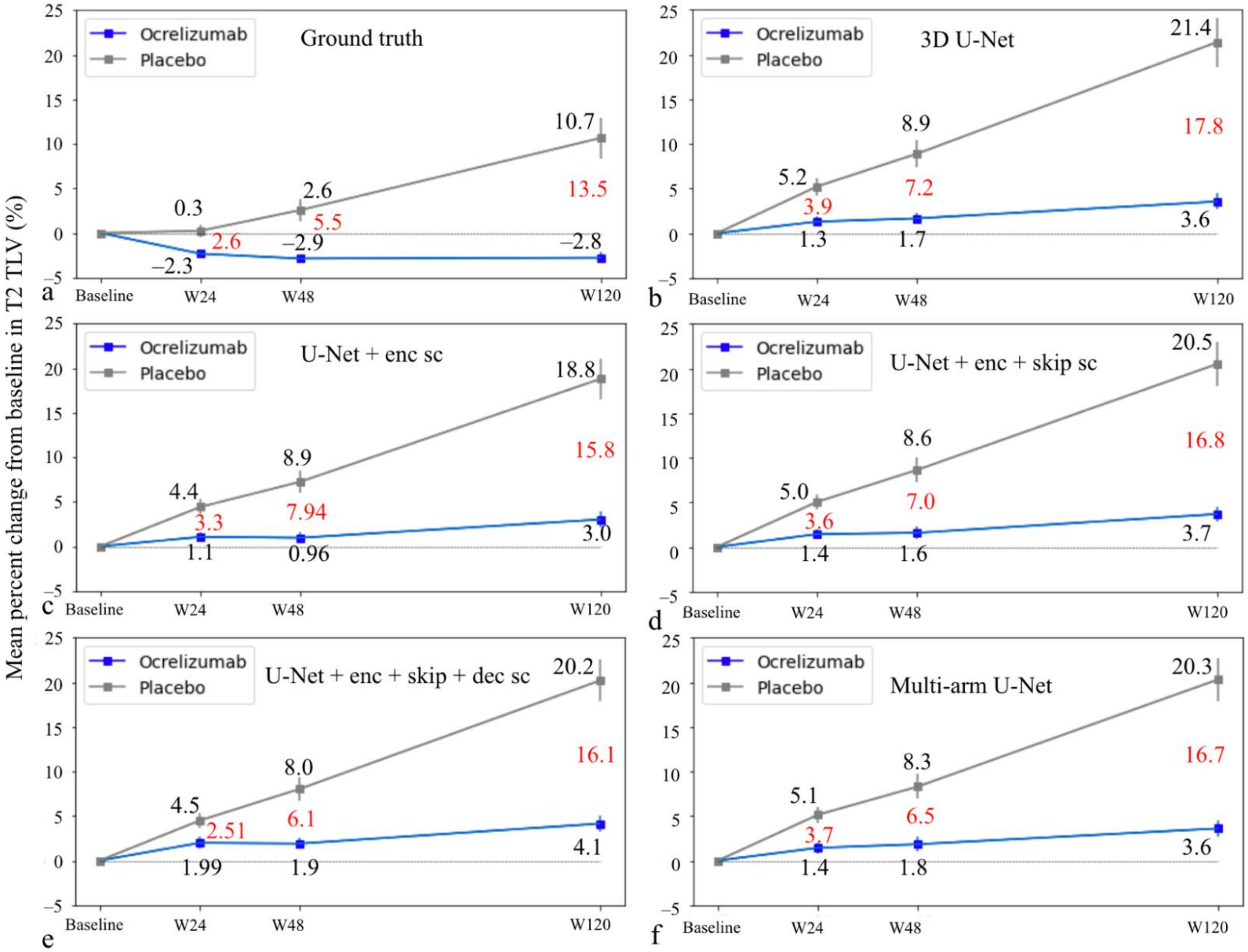

sc – shortcut connections. Percent change in T2 TLV from baseline to Week 120 in the ORATORIO trial using ground truth (GT) masks (a), predicted masks from single-arm U-Net (b), single-arm U-Net with shortcut connections in the encoder(c), single-arm U-Net with shortcut connections in the encoder and skip features (d), single-arm U-Net with shortcut connections in the encoder, skip features and decoder (e) and multi-arm U-Net with all three shortcut connections (f). All models exhibited similar separation between the treatment and placebo arms; the single-arm U-Net with all three shortcut connections had separations closest to those from GT masks; and multi-arm U-Net achieved similar separations with 30% fewer parameters.

Focusing on the smallest lesions (3–10 voxels), the FPR improved slightly with the addition of the various shortcut connections in the single-arm models (0.288, 0.288, 0.274, 0.271). Though we did not assess the multi-arm U-Net without shortcut connections here, such models with attention modules in the skip connections achieved an overall score of ≥ 92.7 in the ISBI 2015 challenge dataset. The addition of shortcut connections improved the score by approximately 0.3%. The mean T2 total lesion volume showed similar separations between the treatment and placebo arms for the different variants. However, the separation of the arms at the follow-up timepoints estimated using the single-arm model with shortcut connections in the encoder, skip features, and decoder had the least difference with the separations estimated from the manual annotations (Figure S3) at the earlier timepoints of Week 24 and 48. Thus, the architectural choices we made for the single-arm U-Net offered a slight benefit for smaller lesions and longitudinal performance; and the multi-arm achieved analogous performance with a reduction in model size. Dec, decoder; enc, encoder; FPR, false positive rate; sc, shortcut connections; TLV, total lesion volume.
